# Influence of experimental protocol on response rate and repeatability of mechanical threshold testing in dogs

**DOI:** 10.1016/j.tvjl.2015.02.008

**Published:** 2015-04

**Authors:** L.K. Harris, J.C. Murrell, E.G.M. van Klink, H.R. Whay

**Affiliations:** School of Veterinary Sciences, University of Bristol, Langford, North Somerset BS40 5DU, United Kingdom

**Keywords:** Canine, Degenerative joint disease, Nociception, Mechanical thresholds, Protocol evaluation

## Abstract

Mechanical threshold (MT) testing is widely used to measure nociceptive thresholds. However, there has been little research into factors that contribute to the response rate and repeatability (collectively termed ‘efficacy’) of MT testing protocols. The aim of this study was to investigate whether the efficacy of a protocol using a hand-held algometer to measure MTs (N) in healthy dogs (*n* = 12) was affected by varying (1) the area over which force was applied (tip diameter), (2) rate of force application, (3) position of dog during testing, and (4) anatomical site of testing. The effect of these factors on MT and the impact of individual dog effects on both efficacy and MT were also investigated.

Overall, 3175/3888 tests (82%) resulted in a measurable response. The response rate was reduced by using wider tip diameters, testing at the tibia, and testing when the dog was lying down (compared to sitting upright). Wider tips were associated with higher, more variable MTs (mean ± standard deviation) with values of 4.18 ± 2.55 N for 2 mm diameter tips, 5.54 ± 3.33 for those of 4 mm, and 7.59 ± 4.73 for 8 mm tips. Individual dog effects had the most significant impact on efficacy and MT. The findings indicate that tip diameter, dog position, and anatomical site may affect both protocol efficacy and MTs, and should be taken into account when comparing different studies and in designing protocols to measure MTs in dogs. The predominant effect of the individual dog over other factors indicates that between-subject differences should always be accounted for in future studies.

## Highlights

• Mechanical nociceptive thresholds were obtained from healthy dogs.
• Effect of protocol on success rate, repeatability and threshold was examined.
• Stimulus area, position of dog and anatomical site affected success rate.
• Wider stimulus areas were associated with higher thresholds.
• Between dog variation had a greater effect than protocol on all three outcomes.

## Introduction

Mechanical threshold testing (MTT) is a method of non-invasively quantifying nociceptive thresholds in awake animals by measuring the magnitude of an increasing mechanical stimulus at which individuals respond (Le Bars et al., 2001). MTT is widely used in veterinary pain and analgesia research (see, for example, Lascelles et al., 1998; Slingsby et al., 2001; Kongara et al., 2009; Vinuela-Fernandez et al., 2011). However, there has been little research into the effect of protocol on the response rate and repeatability (collectively termed ‘efficacy’) of MTT. Previous studies have investigated the effect of protocol on mechanical thresholds (MTs); for example, MTs have been found to differ significantly between different anatomical locations in humans (Johansson et al., 1999), horses (Haussler et al., 2007), and dogs (Coleman et al., 2014). Although the feasibility and repeatability of MTT in dogs have been studied (Briley et al., 2014), the direct effect of protocol on the efficacy of MTT has not been investigated.

Degenerative joint disease (DJD) is highly prevalent in the canine population (Johnston, 1997), and is likely to impact on welfare. Associations between DJD and reduced MT are well established in human medicine (Hendiani et al., 2003; Arendt-Nielsen et al., 2010), and are also evident in dogs (Brydges et al., 2012; Tomas et al., 2014). MTT could be used to measure changes in somatosensory processing associated with DJD, and the effect of treatment; for example, Moss et al. (2007) observed increased MTs in human patients with knee osteoarthritis (OA) following joint mobilisation treatment.

The aim of the current study was to evaluate the effects of tip diameter (the part of the MTT device in contact with the skin), rate of force application, position of dog during testing, and anatomical site of testing on three outcomes: (1) the response rate of MTT (the proportion of tests where an MT could be measured), (2) the repeatability of MTT, and (3) MT. The ultimate aim was to develop a protocol for measuring MTs in dogs with DJD.

## Materials and methods

### Animals

Twelve healthy dogs were studied. They comprised five females (two neutered) and seven males (three neutered) with a mean (range) age and weight of 5.3 (1–13) years, and 20.6 (9–32) kg. Body condition scores (BCS) were 4/9 (*n* = 2), 5/9 (*n* = 8) and 6/9 (*n* = 2). Inclusion criteria were that subjects should not have any illness or injury likely to cause pain or affect normal behavioural responses, or be receiving analgesic medication. The criteria were confirmed by consulting the owners, and informed owner consent was obtained for all dogs.

The study was approved by the University of Bristol Ethical Review Group (UIN number UB/12/005 – 17 February 2012).

### Equipment

MTs were measured in Newtons, using a handheld pressure algometer (ProD-Plus, Topcat Metrology) with interchangeable, hemispherical tips of 2, 4 or 8 mm diameter. The rate of application was kept constant (2 N/s or 4 N/s) by warning lights that turned on if the device changed by 0.5 N/s above or below the set rate.

### Data collection

A single researcher (LKH) carried out all data collection. Before testing, dogs were weighed and assigned a body condition score (Laflamme, 1997). Dogs underwent 12 randomly ordered study sessions (Table 1), one for every combination of protocol factors ‘tip’, ‘rate’ and ‘position’ (sitting or lying) (Table 2). Sessions were divided into three blocks and within each block the algometer was applied once to nine anatomical sites (Table 2) in a randomised order. There was a rest period between blocks to allow at least 15 min between tests at the same site (Dixon et al., 2007). Each session lasted approximately 45–60 min.

All testing was carried out in the same room, in which dogs were familiarised for 5 min before data collection began. Dogs were verbally encouraged to sit or lie down on a fleece mat on the floor. When lying, dogs were positioned in lateral recumbency such that the limb to be tested was dorsal. Dogs were minimally restrained throughout the procedure.

**Fig. 1:**
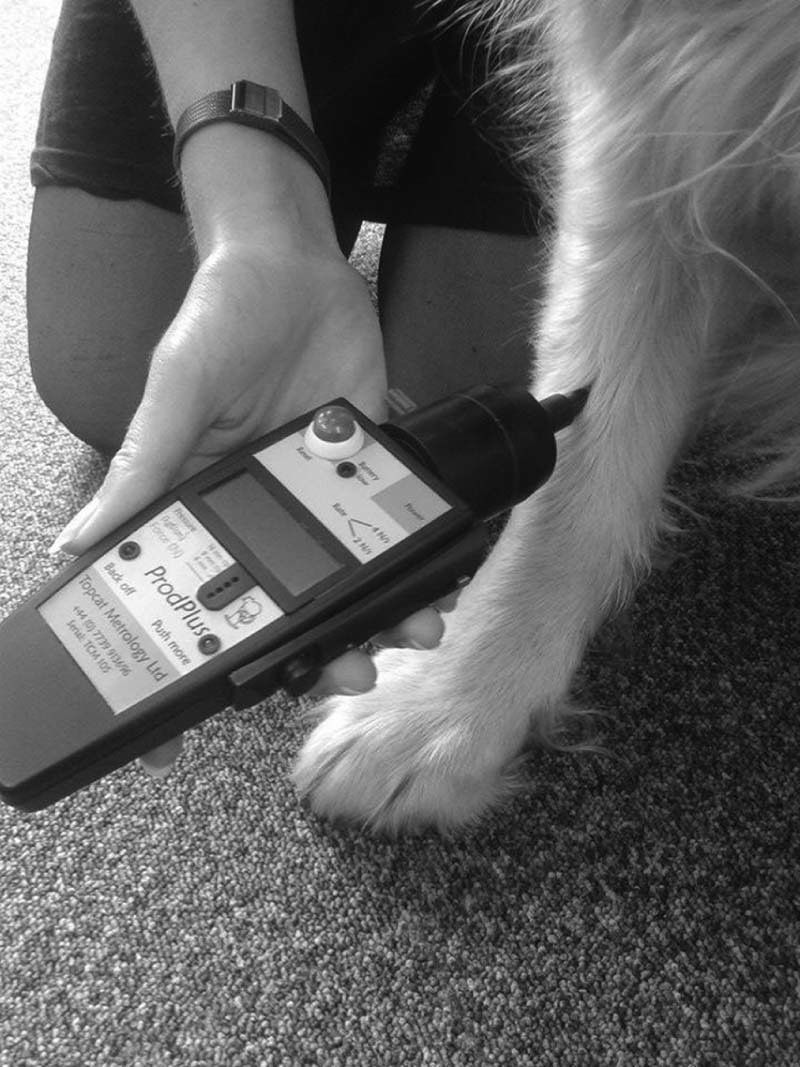
Example of the algometer being applied to the radius of a dog in sitting position.

For each application of the algometer (or ‘test’), the tip was positioned in contact with the anatomical site selected and force was applied by pushing the algometer against the site at a perpendicular angle to the skin surface (Fig. 1). Application of force was immediately stopped if the dog exhibited a clear behavioural endpoint (a deliberate reaction to the stimulus, such as withdrawing the limb). The force at which the animal responded appeared on the algometer screen and was recorded as the MT. If a pre-defined maximum cut-out force (2 mm = 13 N, 4 mm = 15 N, 8 mm = 20 N) was reached before the dog responded, the test was terminated in order to prevent tissue damage, and ‘no response’ was recorded. If an MT could not be obtained for any reason other than reaching the cut-out force, this was recorded as an ‘unmeasurable outcome’ (Table 3).

Depending on availability, most dogs underwent one or two sessions per day, often not consecutively, until all 12 sessions had been completed. Only one dog underwent three sessions in one day. A rest period of at least 1 h was allowed between sessions.

### Data analyses

All analyses were performed using SPSS Statistics version 19.

### Response rate of mechanical threshold testing

A multinomial logistic regression model determined which test factors influenced the likelihood of a measureable response. ‘Tip’, ‘rate’, ‘position’, ‘site’ and ‘dog’ were entered as independent variables and ‘response rate’ was entered as a three-way outcome variable: (1) a measurable response, (2) an unmeasurable outcome and (3) no response. ‘Left radius’ was the reference category for ‘site’ because this category had the greatest proportion of measurable responses.

### Repeatability of mechanical threshold testing

A univariate general linear model (GLM) determined which test factors influenced repeatability of MTT. Repeatability was assessed by calculating the coefficient of variation (CoV) of MTs obtained for each combination of factors ‘tip’, ‘rate’, ‘position’, and ‘site’ within the same subject (‘Dog’). A CoV could only be calculated when two or more MTs had been measured for each combination, but the occurrence of unmeasurable outcomes meant this was not always possible.

‘Tip’, ‘rate’, ‘position’, and ‘site’ were entered as fixed factors, and ‘dog’ as a random factor (Janczak et al., 2012). Standardised residuals of the dependent variable (CoV) were normally distributed after square root transformation and removal of three outliers (±3 standard deviations from the mean) (Shapiro–Wilk's tests: Model 1, *P* = 0.105; Model 2, *P* = 0.174).

### Mechanical thresholds

Kruskal–Wallis tests were used to investigate the effect of each protocol factor, and the effect of ‘dog’, on MT. The factor was entered as the independent variable and the MT as the outcome variable.

## Results

### Response rate of mechanical threshold testing

Overall, 3175/3888 tests (82%) resulted in a measurable response; protocol factors affecting response rate are summarised in Table 4. ‘Dog’ had the greatest effect on response rate, indicated by a strongly significant likelihood ratio, meaning that ‘dog’ contributed significantly to variation in the model (χ^2^ = 372.215, degrees of freedom [df] = 22, *P *<* *0.001). However comparisons between individual dogs were not made due to the small sample size and between-subject variability.

Tests using the 2 mm tip resulted in proportionally less unmeasurable outcomes compared to tests using the 4 and 8 mm tips (2 mm = 15%, 4 mm = 17%, 8 mm = 17%), but these differences were non-significant. The 2 mm and 4 mm tips were both significantly less likely to reach the cut-out force than the 8 mm tip; 2 mm: Wald χ^2^ test (Wald) = 21.680, df = 1, *P *<* *0.001, odds ratio (OR) = 0.145; 4 mm: Wald = 5.002, df = 1, *P* = 0.025, OR = 0.556.

Tests in which the dog was sitting were less likely to result in unmeasurable outcomes than tests where the dog was lying down (Wald = 56.404, df = 1, *P *<* *0.001, OR = 0.493). There was no significant effect of position on the likelihood of a test reaching cut-out. Tests at the right and left tibia, and the right stifle, were significantly more likely to result in unmeasurable outcomes than tests at the left radius (tibia: Wald = 44.592, df = 1, *P *<* *0.001, OR = 3.725 and Wald = 25.697, df = 1, *P *<* *0.001, OR = 2.760, respectively. Right stifle: Wald = 7.663, df = 1, *P* = 0.006, OR = 1.782). Tests at the sternum were more likely to reach cut-out than tests at the left radius (Wald = 4.726, df = 1, *P* = 0.030, OR = 2.945). Rate of force application had no effect on the response rate of the tests (Unmeasurable outcomes: Wald = 2.488, df = 1, *P* = 0.115, OR = 1.156; No response: Wald = 2.976, df = 1, *P* = 0.084, OR = 0.655).

The most common reason for unmeasurable outcomes was avoidance (Table 3). A second logistic regression model was therefore run in which avoidance was separated from other reasons. The position of the dog had a greater effect in the second model compared to the first (Wald = 127.864, df = 1, *P *<* *0.001, OR = 0.258), indicating that tests carried out with the dog in lying position were likely to be unmeasurable due to avoidance.

Testing at the right or left tibia, compared to the left radius, resulted in an increased likelihood that the test would be unmeasurable due to avoidance (right tibia, Wald = 9.374, df = 1, *P* = 0.002, OR = 2.074; left tibia, Wald = 10.214, df = 1, *P* = 0.001, OR = 2.123), but the magnitude of this effect was reduced compared to the combined effect of spontaneous movement and the tip becoming dislodged (right tibia, Wald = 36.228 df = 1, *P *<* *0.001, OR = 10.267; left tibia, Wald = 17.952, df = 1, *P < *0.001, OR = 5.452), indicating that reasons other than avoidance contributed to the high number of unmeasurable outcomes at the tibia. The likelihood that tests at the right stifle would be unmeasurable due to spontaneous movement or the tip being dislodged was significantly greater than tests at the left radius (Wald = 5.487 df = 1, *P* = 0.019, OR = 2.723).

### Repeatability of mechanical threshold testing

There was no significant effect of ‘tip’, ‘rate’, ‘position’ or ‘site’ on CoV. There was a statistically significant overall difference in CoV for repeated tests between different dogs (Table 5).

### Mechanical thresholds

Mechanical thresholds were not influenced by ‘rate’, ‘position’ or ‘site’. Average MTs and standard deviations (SD) increased with tip diameter (2 mm = 4.18 ± 2.55, 4 mm = 5.64 ± 3.33, 8 mm = 7.59 ± 4.73; Kruskal–Wallis: *X^2^* = 328.36, df = 2, *P *<* *0.001). ‘Dog’ had a significant effect on MT (Kruskal–Wallis: *X^2^* = 723.83, df = 11, *P *<* *0.001), and the following between-subject differences were investigated: MTs increased with bodyweight and decreased with age (Spearman's ρ = 0.323, *P *<* *0.001; Spearman's ρ = −0.086, *P *<* *0.001, respectively). Although these correlations were significant, the strength of the associations was low.

Sex alone had no significant effect on MT, but neutered dogs of either sex had significantly higher MT (χ^2^ = 110.06, df = 1, *P *<* *0.001). There was a significant interaction between sex and neuter status, but the number of dogs in each category was small (e.g. neutered females = 2). BCS and breed significantly affected MT (χ^2^ = 262.24, df = 1, *P *<* *0.001; and χ^2^ = 547.10, df = 8, *P *<* *0.001, respectively). However, because of the small number of dogs in each category the biological significance of these findings was unclear.

There was no significant correlation between session order and MT (Spearman's ρ = −0.088, *P* = 0.292).

## Discussion

Factors associated with an MTT protocol have an impact on both the response rate of the test and the MT obtained. The likelihood of a measureable response was significantly decreased by larger tip diameters, dogs lying down rather than sitting upright, and by testing at the tibia compared to other anatomical sites. MTs significantly increased with larger tip diameters. However, the most significant factor affecting MT and response rate, and the only factor to affect repeatability, was ‘dog’.

Poor response rates in MTT lead to missing data points, which can hinder analysis and increase the amount of procedures performed on subjects through repeated tests. Briley et al. (2014) measured the feasibility of MTT by quantifying the degree of cooperation shown by the dogs. Unlike our study, the effects of protocol factors on feasibility were not examined.

The effect of ‘dog’ on response rate could be attributed to differences in individual temperament. Although temperament was not quantified in the current study, it was noted that more hyperactive dogs were likely to avoid application of the algometer. Hyperactive dogs also appeared less willing to lie down, repeatedly rising mid-test; this might, at least potentially, explain the higher response rates when dogs were sitting. Briley et al. (2014) also found that poor cooperation with an MTT procedure led to difficulties in data collection. Flexion of muscles in the hind limb when dogs were sitting made it difficult to access the tibia site, possibly increasing unmeasurable responses due to dislodging. Difficulty in moving away from tests at the sternum, and greater hair coverage at this site, may have contributed to the higher proportion of tests reaching cut-out at the sternum compared to other sites.

The narrowest tip (2 mm) was associated with proportionally less unmeasurable outcomes, and significantly less tests reaching cut-out than wider tips. The lower MTs observed in tests using narrower tips may explain why they were less likely to reach cut-out. These findings suggest that it may be advisable to avoid using wider tips, testing at the tibia, and testing when the dog is lying down in order to increase measurable responses in future MTT protocols.

The decision to test unrestrained animals was made to allow a full range of behavioural responses and to reduce stress. Arguably, if an animal needs to be heavily restrained their responses may not reliably reflect their MT. However, this decision is likely to have contributed to the proportion of unmeasurable outcomes due to avoidance of force application.

Only ‘dog’ and tip diameter had an effect on the MTs we measured. To examine the effect of ‘dog’ on MT, the effects of between-subject differences were investigated. Heavier dogs had higher MTs than lighter dogs, an association that has been observed in previous studies (Moore et al., 2013; Briley et al., 2014). A decrease in mean MT with age has also been reported previously in dogs (Moore et al., 2013), although the effect of ageing on nociception is still unclear (Gibson and Farrell, 2004; Yezierski, 2012).

BCS had an effect on MT in dogs, but the range of BCS in our sample population was 4–6 (out of a range of 1–9), providing low statistical confidence. It is likely that breed and bodyweight interacted; the four dogs with the highest average MTs were all large breed dogs.

The finding that narrower tips were associated with lower MTs, and less variability, which is consistent with previous research in multiple mammalian species, may be attributed to the fact that force is distributed across a smaller area with narrower tips, increasing the pressure on nociceptors in that area (Pressure = force/area) (Taylor and Dixon, 2012).

The lack of influence of anatomical site and rate of force application on MT conflicts with previous research. Human studies have shown differences in MT obtained at different anatomical locations (Johansson et al., 1999). This has been attributed to variation in skin type, for example, glabrous compared to hairy skin (Xiong et al., 2011). In horses, higher MTs were observed at ‘bony’ compared to ‘soft tissue sites’ (Haussler et al., 2007). In the current study all thresholds were obtained at hair covered sites with little subcutaneous tissue, which may explain the similar thresholds observed. However, Coleman et al. (2014) tested similar anatomical sites in healthy dogs and found significant differences. This incongruence may be attributable to sample differences; Coleman et al. (2014) tested 19 dogs, all of which were retriever type breeds; our sample was smaller and less homogeneous, which possibly made any site differences statistically non-significant compared to the effects of between subject differences.

An association between rate and MT has been observed in donkeys (Grint et al., 2014). The dogs in the current study were less homogeneous as a group, compared to the donkeys, which may have masked the effect of rate. Variation in between-subject differences may also explain why individual dog was the only factor to significantly affect repeatability.

The algometer used in this study was chosen because it is easy to use and does not require a Home Office licence when used in a clinical setting (Hunt et al., 2013). A drawback of hand-held algometers is that they are more likely to become dislodged than limb-mounted devices (Nalon et al., 2013). Previous MTT studies have used limb-mounted algometers (Millette et al., 2008; Dixon et al., 2010; Hothersall et al., 2011); however, these would not have been suitable in the current study as they cannot be securely attached to joints.

Lack of comparable studies meant that there was insufficient information to calculate adequate sample size; 12 dogs are a relatively small sample, which may not be representative of the population. The repeated measures study design meant that there was sufficient data to carry out robust statistical analysis of the effect of within-subject factors (i.e. protocol factors) but not necessarily between-subject factors (i.e. demographic differences such as bodyweight and age). Further studies with larger sample sizes are recommended.

## Conclusions

This study indicated that tip diameter, position of dog during testing and anatomical site of testing may impact on the efficacy of MTT when a single researcher is performing tests on unrestrained dogs. It is recommended that a 2 mm tip be used with the subject in the sitting position, and that testing at the tibia is avoided when using this algometer. Tip diameter should be taken into account when comparing findings from different studies as it is likely to affect MTs. Between-subject differences may influence efficacy and MT; when comparing groups, dogs should be matched on the basis of weight, sex (and neuter status) and age. A quantification of temperament would be a useful addition to future research. It is hoped that the knowledge obtained in this study will help to establish an optimal protocol for measuring changes in MT in dogs with DJD, which may provide a reliable, objective method for assessing somatosensory changes associated with disease progression or response to treatment.

## Conflict of interest statement

None of the authors have any financial or personal relationships that could inappropriately influence or bias the content of the paper.

## Figures and Tables

**Table 1:** Summary of a typical session. Dogs underwent 12 sessions, each with a different combination of tip, rate and position. The order of sessions was randomised for each dog, and the order in which the sites were tested was randomised for each block.

Session 1 (tip = 2 mm, rate = 4 N/s, position = sitting)							
Block 1			Block 2			Block 3	
Test number	Site		Test number	Site		Test number	Site
1	Right radius		10	Right elbow		19	Right tibia
2	Left radius		11	Left radius		20	Right elbow
3	Left stifle		12	Sternum		21	Right radius
4	Left elbow		13	Left tibia		22	Left elbow
5	Sternum	15 min rest	14	Right radius	15 min rest	23	Sternum
6	Right elbow		15	Left stifle		24	Left stifle
7	Right stifle		16	Right tibia		25	Left tibia
8	Left tibia		17	Right stifle		26	Left radius
9	Right tibia		18	Left elbow		27	Right stifle

**Table 2:** Summary of average mechanical thresholds (MTs).

Factor		Average MT – all 12 dogs included (mean ± SD)
Rate	2 N/s	5.8 ± 4.0
	4 N/s	5.8 ± 3.8
Tip	2 mm	4.18 ± 2.55^a^
	4 mm	5.64 ± 3.33^a^
	8 mm	7.59 ± 4.73^a^
Position	Sitting – *upright posture, hind quarters lowered.*	5.7 ± 3.9
	Lying – *lateral recumbency*	5.8 ± 3.9
Site	Right radius – *midpoint along the length of the right radius, dorsal aspect*	6.0 ± 4.1
	Left radius – *midpoint along the length of the left radius, dorsal aspect*	5.7 ± 4.0
	Right elbow – *lateral condyle of the right humerus*	5.7 ± 4.3
	Left elbow – *lateral condyle of the left humerus*	5.7 ± 3.9
	Right tibia – *midpoint along the length of the right tibia, lateral aspect*	5.6 ± 3.8
	Left tibia – *midpoint along the length of the left tibia, lateral aspect*	5.8 ± 3.8
	Right stifle – *lateral condyle of the right femur*	5.6 ± 4.0
	Left stifle – *lateral condyle of the left femur*	5.4 ± 3.7
	Sternum – *proximal sternum, at the point where the forelimbs join the torso.*	6.3 ± 3.8

a Tip diameter had a significant effect on MT (larger tips were associated with higher MT) *P *<* *0.05.

**Table 3:** List of reasons for unmeasurable outcomes, descriptions and proportions of unmeasurable outcomes attributed to these reasons. Final row details criteria for the ‘no response’ outcome.

Reason	Description	Proportion of unmeasurable outcomes attributed to this reason (number of tests unmeasurable for this reason/total unmeasurable tests)
Avoidance	Dog was actively evading application of the algometer (including withdrawal of limb at the touch of the algometer, before force application began)	461/638 (~72.2%)
Dislodged	The tip slipped from the anatomical site after force application had begun but before a behavioural response was observed.	123/638 (~19.3%)
Spontaneous movement	The dog performed a behaviour that was not an obvious reaction to the stimulus (e.g. the dog started grooming.)	54/638 (~8.5%)
No response (cut-out force reached)	The predefined maximum cut-out force was reached before a behavioural response was observed (maximum cut out forces were set to avoid tissue damage and were based on whether application to human skin at this force left a visible mark for more than 1 min: 2 mm = 13 N, 4 mm = 15 N, 8 mm = 20 N)	75

**Table 4:** Logistic regression model showing the effect of protocol factors on response rate of tests performed. As the number of tests is a discrete number, percentages are rounded to the nearest whole number (≥0.5 = round up); as a result, some percentages may not add up to 100% where expected.

		Number of tests (*n*)	Measurable responses	Unmeasurable outcome			No response		
			Proportion of tests (%)	%	Odds ratio (OR)	Confidence interval (CI)	%	Odds ratio (OR)	Confidence interval (CI)
Overall (all tests)		3888	82	16	–	–	2	–	–
Rate	2 N/s	1944	81	17	1.156	0.965–1.385	2	0.655	0.405–1.059
	4 N/s	1944	82	16	Ref.^c^	–	2	Ref.^c^	–
Tip	2 mm	1296	85	15	0.825	0.660–1.032	1	0.145	0.064–0.327
	4 mm	1296	81	17	1.031	0.830–1.281	2^a^	0.556	0.333–0.930
	8 mm	1296	80	17	Ref.^c^	–	3	Ref.^c^	–
Position	Sitting	1944	86	12^b^	0.493	0.410–0.593	2	1.274	0.790–2.053
	Lying	1944	78	21	Ref.^c^	–	2	Ref.^c^	–
Site (R = right, L = left)	Sternum	432	84	12	1.179	0.765–1.819	4^a^	2.945	1.112–7.801
	R. stifle	432	81	17	1.782	1.184–2.683	2	1.624	0.560–4.714
	L. stifle	432	85	15	1.431	0.941–2.176	1	0.675	0.186–2.457
	R. tibia	432	69	29^b^	3.725	2.532–5.480	2	1.823	0.628–5.295
	L. tibia	432	76	24^b^	2.760	1.864–4.087	1	0.735	0.202–2.674
	R. elbow	432	83	14	1.421	0.933–2.165	3	1.972	0.705–5.513
	L. elbow	432	84	13	1.281	0.836–19.63	3	2.149	0.780–5.923
	R. radius	432	86	13	1.261	0.823–1.932	1	0.668	0.184–2.429
	L. radius	432	88	11	Ref.^c^	–	1	Ref.^c^	–

a *P* < 0.05 significance of effects of test factors on likelihood of a test resulting in an unmeasurable outcome or no response compared to a measurable response (i.e. an MT obtained).

b *P* < 0.001 significance of effects of test factors on likelihood of a test resulting in an unmeasurable outcome or no response compared to a measurable response (i.e. an MT obtained).

c Ref., reference. Last category was selected as the reference category for each variable (apart from Site; in this case, left radius was chosen as the reference category because it had the highest response rate).

**Table 5:** Main effects and significant two way interactions of a univariate general linear model: effects of protocol related factors on repeatability of a mechanical threshold test (represented by coefficient of variance, CoV).

Source	DF (hypothesis)	DF (error)	F^a^	*P*^b^	η^2^
Rate	1	11.763	0.037	0.850	0.003
Tip	2	22.963	1.490	0.246	0.115
Position	1	11.733	0.843	0.377	0.067
Site	8	90.639	0.745	0.651	0.062
Dog	11	12.413	4.000	0.011*	0.780
Site × dog	88	933	1.548	0.001**	0.127

DF, degrees of freedom; F, the F statistic; *P*, the statistical significance of the effect; η^2^, the partial eta-squared statistic (the proportion of variability in CoV attributable to the factor).

a Test of the null hypothesis that the factor has no effect on CoV.

b Effect considered significant at *P *<* *0.05 (*), or *P* < 0.001 (**).
